# Platinum response characteristics of patients with pancreatic ductal adenocarcinoma and a germline *BRCA1, BRCA2* or *PALB2* mutation

**DOI:** 10.1038/s41416-019-0582-7

**Published:** 2019-12-02

**Authors:** Max M. Wattenberg, Daniella Asch, Shun Yu, Peter J. O’Dwyer, Susan M. Domchek, Katherine L. Nathanson, Mark A. Rosen, Gregory L. Beatty, Evan S. Siegelman, Kim A. Reiss

**Affiliations:** 1 Division of Hematology-Oncology, Department of Internal Medicine, Perelman School of Medicine, University of Pennsylvania, Philadelphia, PA USA; 2 Department of Radiology, Perelman School of Medicine, University of Pennsylvania, Philadelphia, PA USA; 3 Basser Center for BRCA, Abramson Cancer Center, University of Pennsylvania, Philadelphia, PA USA

**Keywords:** Pancreatic cancer, Cancer genetics

## Abstract

**Background:**

Retrospective studies suggest a survival benefit when platinum-based chemotherapy is administered to patients with pancreatic cancer harbouring a germline mutation in *BRCA1, BRCA2* or *PALB2* (mut-positive PDAC). However, the objective response rate (ORR) and real-world progression free survival (rwPFS) achieved with such treatment remain ill-defined.

**Methods:**

Twenty-six patients with advanced-stage mut-positive PDAC who had been treated with platinum-based therapy were matched by age, race and sex to 52 platinum-treated control PDAC patients. Responses to therapy were determined by RECIST v1.1, performed by blinded radiology review. Measured outcomes included ORR and rwPFS.

**Results:**

The ORR in mut-positive patients was 58% compared to 21% in the control group (*p* = 0.0022). There was no significant difference in ORR between platinum regimens in mut-positive patients (*p* = 0.814), whereas in control patients, the only observed responses were to FOLFIRINOX. rwPFS was 10.1 mo. for mut-positive patients and 6.9 mo. for controls (HR 0.43; 95% CI 0.25–0.74; 0.0068).

**Conclusion:**

Mut-positive PDAC has a high ORR and prolonged rwPFS to platinum-based chemotherapy. These findings may have implications particularly in the neoadjuvant setting, and for future clinical trial design, and highlight the importance of early germline testing in patients with PDAC.

## Background

Pancreatic ductal adenocarcinoma (PDAC) is a lethal malignancy with a median overall survival of less than one year for patients with advanced disease.^1–3^ Historically, there have been no predictive biomarkers to discriminate treatment choice in pancreatic cancer. However, unique biological subsets of PDAC with distinctive clinical characteristics, including sensitivity to specific therapies, have become increasingly recognised.^4^ One such subgroup includes carriers of a mutation in one of the homologous recombination genes *BRCA1, BRCA2* or *PALB2*. This phenomenon occurs in approximately 6% of the overall PDAC population, with higher rates in those with a personal or family history of *BRCA*-related malignancies.^5–10^

BRCA1 and BRCA2 are key proteins involved in homologous recombination,^11,12^ and PALB2 is an essential regulator of BRCA2 function.^13^ Loss of function of these genes leads to homologous recombination deficiency (HRD). Due to the inability to effectively repair double strand DNA breaks, tumours that harbour a HRD are particularly sensitive to DNA damaging and cross-linking agents such as platinum-based chemotherapy.^14,15^ Clinically, breast and ovarian cancers associated with *BRCA1* and *BRCA2* mutations show heightened sensitivity to platinum-based chemotherapy.^16,17^

Tailored treatment strategies for patients with *BRCA- or PALB2-*associated PDAC are actively being studied and refined, particularly regarding platinum therapy and PARP inhibitors. We and others have previously shown that platinum-based chemotherapy improves overall survival in patients with advanced PDAC harbouring a germline mutation in *BRCA1, BRCA2* or *PALB2*.^18–20^ In addition, while prior studies have suggested high response rates to platinum-based chemotherapy,^21^ response characteristics have not been robustly defined in this subgroup. Response characteristics have implications for the choice of pre-operative therapy in the management of locally advanced or borderline resectable disease and as a benchmark for future clinical trial design. As neoadjuvant therapies become more commonly incorporated into the treatment of non-metastatic PDAC patients, knowledge of the overall response rate to specific treatments will be increasingly important to guide patient care and to provide a basis for further treatment advances.

Here, we performed a retrospective cohort study of patients with PDAC who had been treated with platinum-based chemotherapy. We aimed to understand both the degree and the durability of response to platinum-based chemotherapy in patients with PDAC harbouring a pathogenic germline mutation in *BRCA* or *PALB2*, compared to those without such a mutation.

## Methods

### Patient identification and data collection

A single centre, retrospective analysis of patients with PDAC was performed. Patients diagnosed with PDAC who had received platinum-based therapy between 1 July 2011 and 30 March 2018 and for whom serial cross-sectional imaging files were available were included. ‘Mut-positive’ patients were those with a known pathogenic germline *BRCA1, BRCA2* or *PALB2* mutation. ‘Controls’ were either confirmed mutation non-carriers or those who had not been tested. Collected data included demographics and treatment history. Each mut-positive patient was matched to two controls using a greedy-match algorithm^22^ without replacement and was based on three patient factors: (1) age at diagnosis, (2) sex and (3) race. During matching, age at diagnosis was assigned the most weight, followed by gender and race. Each case was successfully matched to two eligible controls patients.

Baseline imaging was defined as the cross-sectional body imaging study performed closest to the start date of platinum-based therapy and no more than 28 days from this date. Serial restaging imaging studies were gathered for each patient. All imaging studies were uploaded into MINT version 3.4.3 (Mint Medical GMBH, Heidelberg, Germany) for analysis and were evaluated by a radiologist who was blinded to patient mutational status and clinical history. A second radiologist who was also blinded to the same variables confirmed all radiology reads. The institutional review board at the University of Pennsylvania approved this study.

### Response evaluation

Responses were determined using RECIST v1.1. Overall response rate (ORR) was defined as the percentage of patients who had a best-response rating of complete response (CR) or partial response (PR) at any time point while on treatment with platinum-based chemotherapy. Patients without measurable disease at baseline, such as those with non-measurable locally advanced disease, were not included in the ORR analysis. Disease control rate (DCR) was defined as the percentage of patients who had a best-response rating of CR, PR or stable disease (SD) at any time point while on treatment with platinum-based chemotherapy. Patients without measurable disease at baseline were defined as having stable disease or progressive disease based on evaluation of non-target lesions and were included in the DCR analysis.

### Real-world progression free survival

Real-world progression free survival (rwPFS) was defined as the number of days between the date of first platinum chemotherapy administration and the date of clinical platinum failure or death.^23^ Platinum failure was defined as any time point at which the treating oncologist either switched or stopped platinum therapy due to disease progression. Discontinuation of platinum due to toxicity, due to a switch to maintenance treatment, or due to ability of the patient to receive local therapy (i.e. surgical resection) was not considered to be platinum failure. If a patient was not known to have switched or discontinued treatment due to progression and was not known to have died, the rwPFS was censored at the last date of follow-up. The cutoff date was 31 December 2018.

### Overall survival

Overall survival (OS) was defined as the number of days between the date of diagnosis of advanced disease and the date of death from any cause. If a patient was not known to have died, the OS was censored at last date of follow-up. The cutoff date was 31 December 2018.

### Statistical analysis

Univariate analysis was performed using Student’s *t*-test or Wilcoxon rank-sum test for normal and non-normal continuous variables, respectively, and the chi-square test was used for categorical variables. Both tests were used with a two-sided alpha of 0.05. The Kaplan-Meier methodology was used to calculate the median rwPFS and OS for each group and comparison of survival curves was performed using the log-rank (Mantel-Cox) test. The effect size was estimated using the log-rank test and was reported as a hazard ratio with 95% confidence intervals. Statistical analysis was performed using Prism 8.0 software (GraphPad, San Diego, CA).

## Results

### Patient characteristics

Overall, 26 mut-positive patients with either locally advanced or metastatic PDAC were identified with the following mutational breakdown: *BRCA1* (*n* = 5), *BRCA2* (*n* = 17) and *PALB2* (*n* = 4). Patients who were mut-positive were matched with 52 control patients as described in the Methods. The mean age of mut-positive patients was 59 years (range: 41–81). The mean age of control patients was 60 years (range: 47–82). There were seven black patients in the control group and none in the mut-positive group (*p* = 0.049). The year at start of treatment was similar between mut-positive (median, 2016) and control patients (median, 2017) but the difference did meet statistical significance (*p* = 0.044). All patients had either locally advanced or metastatic disease at start of platinum-based therapy. A numerically higher percentage of patients in the mut-positive group had metastatic disease at start of platinum-based treatment (85% vs. 67%; *p* = 0.104). Both groups had a similar number of metastatic sites of disease. Mut-positive patients were more likely to have metastatic disease to the liver at time of platinum-based treatment initiation (*p* = 0.024) (Table 1).

**Table 1:** Demographic and baseline characteristics

Characteristic	Controls (*N* = 52)	Mut-positive (*N* = 26)	*P*-value
Age at diagnosis—year			
Mean	60	59	0.83
Range	47–82	41–81	–
Sex—no. (%)			
Male	27 (52)	11 (42)	0.42
Female	25 (48)	15 (58)	
Race or ethnic group—no. (%)^a^			
Asian	3 (6)	1 (4)	0.76
Black	7 (13)	0 (0)	0.049
White	42 (81)	25 (96)	0.065
Year at initiation of treatment			
Median	2017	2016	0.044
Range	2014–2018	2013–2018	–
Stage—no. (%)			
Locally advanced	17 (33)	4 (15)	0.104
Metastatic	35 (67)	22 (85)	
No. of metastatic sites involved			
Median	1	1	0.36
Range	0–3	0–3	–
Location of metastatic sites—no. (%)			
Liver	24 (46)	19 (70)	0.024
Lung	7 (13)	3 (11)	0.81
Lymph node	11 (21)	4 (21)	0.54
Other	10 (19)	4 (15)	0.68
Germline mutation—no. (%)			
BRCA1		5 (19.2)	–
BRCA2		17 (65.4)	–
PALB2		4 (15.4)	–

A greater number of black patients were included in the control group (*p* = 0.0499 by the Chi-square test for comparing proportions)

^a^Race was reported by the investigator

### Clinical and treatment characteristics of patients

All patients received platinum-based chemotherapy for advanced PDAC. The regimens used for mut-positive patients were FOLFIRINOX (*n* = 10; 38.5%), FOLFOX (*n* = 10; 38.5%) and cisplatin plus gemcitabine (*n* = 6; 23.0%). One patient received FOLFIRINOX followed by cisplatin plus gemcitabine, switching due to an allergic reaction and severe hand-foot syndrome from 5-FU therapy. The regimens used in control patients were FOLFIRINOX (*n* = 39; 75%), FOLFOX (*n* = 11; 21.1%), cisplatin plus gemcitabine (*n* = 1; 1.9%) and cisplatin plus gemcitabine plus nab-paclitaxel (*n* = 1; 1.9%). Platinum therapy was most commonly received in the first-line setting regardless of cohort: 80.7% of mut-positive patients received platinum in the first-line, as did 67.3% of control patients (*p* = 0.21). Significantly more control patients received FOLFIRINOX (75% vs. 38.5%; *p* = 0.0016) and significantly more mut-positive patients received cisplatin plus gemcitabine (23.1% vs. 1.9%; *p* = 0.0021) (Table 2).

**Table 2:** Treatment characteristics

Prior therapy	Controls (*N* = 52)	Mut-positive (*N* = 26)	*P*-value
Platinum-based regimen—no. (%)			
FOLFIRINOX	39 (75)	10 (38)	0.0016
FOLFOX	11 (21)	10 (38)	0.1
Gemcitabine cisplatin	1 (2)	6 (23)	0.0021
Gemcitabine cisplatin nab-paclitaxel	1 (2)	0 (0)	0.476
Line of therapy containing platinum—no. (%)			
First	35 (67)	21 (81)	0.21
Second	15 (29)	4 (15)	0.19
Third	2 (4)	1 (4)	>0.9

FOLFIRINOX includes fluorouracil, leucovorin, irinotecan and oxaliplatin

FOLFOX includes fluorouracil, leucovorin and oxaliplatin

One patient in the mut-positive group received FOLFIRINOX followed by gemcitabine cisplatin; the change was made due to 5-FU induced hand-foot syndrome

### Overall response rate

**Fig. 1:**
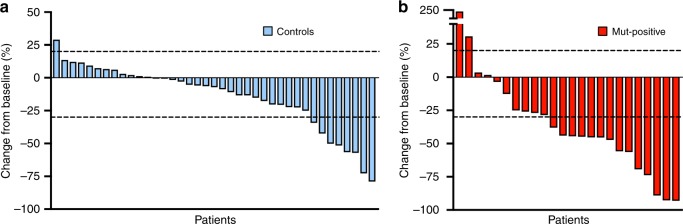
Best Objective Response to Platinum Based Treatment. Waterfall plots showing best percentage change in target-lesion size from time of initiation of platinum-based therapy. **a** Best overall change in tumour size in control patients. For the control cohort, 13 patients were not included due to undefined change in tumour size. **b** Best overall change in tumour size in mut-positive patients. For the mut-positive cohort, two patients were not included due to undefined change in tumour size. The upper dashed line indicates a 20% increase in tumour size and the lower dashed line indicates a 30% reduction in tumour size

Two mut-positive patients and 13 control patients had no measurable disease at baseline and were excluded from the overall response rate (ORR) analysis. The ORR as defined by RECIST v1.1, was significantly higher for mut-positive patients as compared to control patients (58% vs. 21%; *p* = 0.0022) (Fig. 1). For mut-positive patients, partial response (PR) and stable disease (SD) were seen in 58% (*n* = 14) and 21% (*n* = 5) of patients, respectively. None of the mut-positive patients achieved a complete response (CR). Five mut-positive patients (21%) had progressive disease (PD) as best response. For control patients, PR and SD were seen in 18% (*n* = 7), and 62% (*n* = 24), respectively. One (2%) control patient achieved a CR. For mut-positive patients with measurable disease at baseline, the ORR for patients treated with FOLFIRINOX was 60% (*n* = 10); 50% for those treated with FOLFOX (*n* = 8); and 66.7% for those treated with cisplatin plus gemcitabine (*n* = 6). Overall, there was no significant difference in ORR between platinum-based chemotherapy regimens in the mut-positive patients (*p* = 0.814). All objective responses in the control group occurred in the FOLFIRINOX treated patients. All enrolled patients were included in the disease control rate (DCR) analysis. Patients without measurable disease at baseline were defined as having SD or PD based on non-target lesions. The DCR was 81% in both cohorts (*p* > 0.99) (Table 3).

**Table 3:** Summary of efficacy measures

Outcome	Controls (*N* = 52)	Mut-positive (*N* = 26)	*P*-value
Overall survival (mo)			0.0467
Median	18.8	24.6	–
Real-world progression free survival (mo)			0.0068
Median	6.9	10.1	–
Overall response rate—no. (%)^a^	8 (21)	14 (58)	0.0022
Complete response	1	0	–
Partial response	7	14	–
Stable disease	24	5	–
Progressive disease	7	5	–
Disease control rate—no. (%)^b^	42 (81)	21 (81)	> 0.99
Overall response rate by regimen - no. with response/no. evaluable (%)^a^		–	
FOLFIRINOX	8/29 (28)	6/10 (60)	–
FOLFOX	0 (0)	4/8 (50)	–
Gemcitabine cisplatin	0 (0)	4/6 (67)	–

Response criteria according to RECIST (response evaluation criteria in solid tumours) v1.1

Patients in the control group (*n* = 13) and mut-positive group (*n* = 2) without measurable disease at baseline were excluded from overall response rate analysis. The two patients in the mut-positive group without measurable were treated with FOLFOX and were thus excluded from the subset analysis of overall response to FOLFOX

^a^The overall response rate was defined as the percentage of patients that achieved complete response or partial response

^b^The disease control rate was defined as the percentage of patients that achieved complete response, partial response or stable disease. Patients without measurable disease at baseline were included in the DCR analysis. Patients were defined as having SD or PD based on evaluation of non-target lesions

A subset analysis was performed to test the impact of line of therapy on ORR. Mut-positive patients who received platinum-based therapy in the first-line setting had a significantly higher ORR than control patients who received platinum in the first-line setting (68% vs. 29%; *p* *=* 0.007) and a numerically higher ORR when compared to mut-positive patients who received platinum in the second-line setting (68% vs. 20%; *p* = 0.0507). There were no responses in the control group when these patients received platinum in the second-line or later (Table 4).

**Table 4:** Efficacy Measures Stratified by Line of Therapy

Outcome	Controls (*N* = 52)	Mut-positive (*N* = 26)	*P*-value
Real-world progression free survival by line of therapy (mo)			
First	7.9	21.1	0.0046
Second or later	3.4	2.5	0.43
Overall response rate by line of therapy—no. with response/no. evaluable (%)			
First	8/28 (29)	13/19 (68)	0.007
Second or later	0/11 (0)	1/5 (20)	0.12

A subset analysis to compare ORR and rwPFS by line of therapy in which platinum-based treatment was initiated was performed

### Real-world progression free survival

**Fig. 2:**
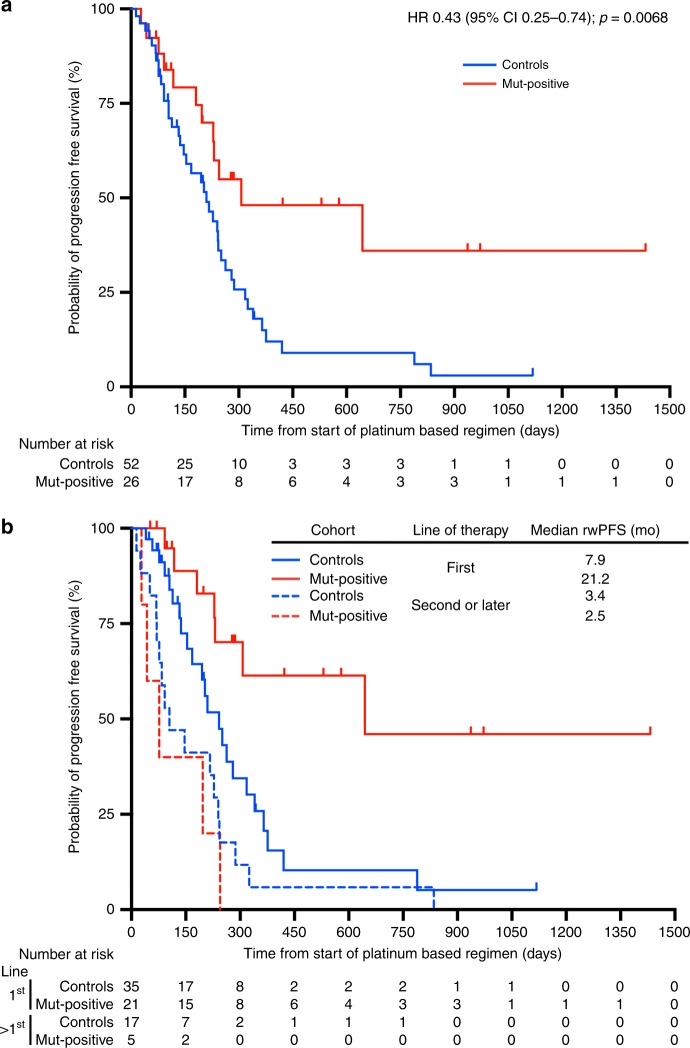
Real World Progression Free Survival. **a** The rwPFS was 10.1 months in the mut-positive cohort as compared to 6.9 months in the control cohort (HR 0.43; 95% CI 0.25–0.74; *p* = 0.0068). **b** A subset analysis comparing rwPFS with platinum use, by line of therapy. The rwPFS of mut-positive patients who received platinum-based chemotherapy in the first-line setting was 21.2 months as compared to 7.9 months for control patients treated with platinum in the first-line setting (*p* *=* 0.0046) and 2.5 months for mut-positive patients treated with platinum in the second-line setting (*p* *=* 0.0001). Kaplan-Meier methodology was used to estimate rwPFS. The hazard ratio was estimated by means of the log-rank test

As a clinically relevant surrogate of durability of platinum response, we utilised rwPFS, defined as the date of first platinum chemotherapy administration for advanced disease to the date of clinical treatment failure or death. Mut-positive patients had a significantly longer rwPFS as compared to control patients (hazard ratio 0.43; 95% confidence interval [CI] 0.25–0.74; *p* = 0.0068). The median rwPFS was 10.1 months for mut-positive patients and 6.9 months for control patients (Fig. 2a). A subset analysis was performed to test the impact of line of therapy on rwPFS. Mut-positive patients treated with platinum in the first-line had the highest rwPFS of 21.1 months as compared to 7.9 months for control patients treated in the first-line (*p* *=* 0.0046) and 2.5 months for mut-positive patients treated with platinum in the second-line or later (0.0001). Control patients treated with platinum in the second-line or later had a rwPFS of 3.4 months (Fig. 2b).

### Overall survival

The OS for mut-positive patients was 24.6 months and 18.8 months for control patients (*p* *=* 0.0467) (Table 3 and Supplementary Fig. 1).

## Discussion

In several prior retrospective studies, it has been suggested that overall survival is improved when platinum-based therapy is administered to patients with pancreatic cancer harbouring germline *BRCA* or *PALB2* mutations.^18,19,24^ However, less is known about the objective response rate to these drugs in this subgroup, and this knowledge may help guide treatment decisions in the neoadjuvant setting and for symptomatic patients, as well as inform clinical trial design for this group. We conducted a case-cohort study to further evaluate this issue. Our primary goal was to understand both the degree and durability of responses to platinum-based chemotherapy in this subgroup of pancreatic cancer patients.

Using blinded radiology review, we analysed the overall response rate (ORR) to platinum-based chemotherapy in matched mutation carriers and control patients in order to determine the degree of response in each cohort. For this analysis, we utilised RECIST v1.1 criteria and only included patients with measurable disease at baseline. We observed an ORR of 58% for mut-positive patients compared to 21% for patients without a known mutation (control) (*p* = 0.0022). FOLFIRINOX was the most commonly used regimen in control patients (75% vs. 38.5%; *p* = 0.0016) while significantly more mut-positive patients received cisplatin plus gemcitabine (23% vs. 2%; *p* = 0.0021). FOLFIRINOX was also the only platinum regimen that resulted in responses in the control group, whereas we observed responses across all platinum regimens in mut-positive patients.

We then performed a subset analysis evaluating ORR to each specific regimen used within mut-positive patients. Although not statistically significant, ORR was highest in those receiving cisplatin plus gemcitabine (66.7% ORR) compared to FOLFIRINOX (60% ORR) and FOLFOX (50% ORR). It is notable that for each regimen, the ORR in mut-positive patients was higher than the published ORR of FOLFIRINOX, providing further evidence that patients with *BRCA* or *PALB2* mutation-associated PDAC are specifically sensitive to platinum treatments. This is consistent with data in other *BRCA*-associated malignancies.^16,17^ Additionally, despite significantly more control patients receiving FOLFIRINOX, the ORR to platinum therapy still favoured the mut-positive cohort, suggesting that the inclusion of a platinum agent, regardless of regimen intensity, may be the key factor in determining response rate in this biologically distinct group of patients.

In our second analysis, we used real-world progression free survival (rwPFS) as a surrogate for durability of platinum response.^23^ Here, we included all patients regardless of measurable disease status. rwPFS was calculated using the time from start of platinum treatment to either platinum failure or death, as identified by investigator review of clinician notes and radiologic reports. We observed a rwPFS of 10.1 months for mut-positive patients compared to 6.9 months for control patients (*p* = 0.0068), highlighting the heightened durability of platinum-based therapy in mut-positive patients.

There are several important limitations to this small, single institution, retrospective analysis. First, two patients (8%) in the mut-positive group and 13 (25%) in the control group were without measurable disease at baseline. These patients were excluded from the ORR analysis but included in the rwPFS analysis. A larger number of patients in the control cohort without measurable disease at baseline would be expected to bias towards the null hypothesis. Second, although we found no significant difference in the number of metastatic sites or stage between groups, mut-positive patients were statistically more likely to have liver predominant metastatic disease (*p* = 0.024) and were numerically more likely to have metastatic disease at study start (85% vs. 67%; *p* = 0.104). Both findings would plausibly bias toward the null hypothesis.^25^ Third, we found that more patients in the mut-positive group received platinum-based chemotherapy in the first-line setting (80.7% vs. 67.3%; *p* = 0.21). Although this finding was not statistically significant, earlier administration of active chemotherapy may favour the mut-positive group in this analysis. Given the potential imbalance between groups in regard to line of therapy in which platinum was delivered, we conducted a subset analysis to understand the impact of line of therapy on ORR and rwPFS. When the analysis was restricted to patients treated with platinum in the first-line setting, ORR and rwPFS remained significantly increased in the mut-positive group as compared to control patients. However, there was no difference in outcomes between groups when platinum was administered in the second-line or later. Additionally, mut-positive patients appeared to derive substantially greater benefit from platinum-based chemotherapy when this was delivered in the first-line with a rwPFS of 21.1 months as compared to 2.5 months when platinum was delivered in the second-line or later (*p* *=* 0.0001).

Our findings have several clinical implications. The high ORR of 58% to platinum therapy in mut-positive patients suggests that platinum therapy may be particularly desirable for this subset of patients in clinical scenarios marked by high disease burden, symptomatic disease and for patients with locally advanced or borderline resectable tumours. As responses were seen regardless of the platinum backbone, our findings also suggest that for mut-positive patients unable to tolerate FOLFIRINOX, alternative platinum-based therapies should be considered.

The National Comprehensive Cancer Network and American Society of Clinical Oncology guidelines now include recommendations for germline testing in all patients with newly diagnosed PDAC, given the treatment implications for this group.^26,27^ We found that patients with *BRCA* or *PALB2* mutant-associated pancreatic cancer may derive greater benefit from initiation of platinum-based chemotherapy in the first-line setting. Taken together, our findings support the need for upfront testing in all patients in order to select the optimal treatment.

Additionally, our findings may help inform future clinical trial design for patients with pancreatic cancer. The ORR and rwPFS reported in this study may provide guidance as a benchmark in future studies evaluating patients with germline *BRCA* or *PALB2* mutation-associated PDAC. Additionally, for clinical trials evaluating platinum-based chemotherapy in all-comers with pancreatic cancer, special attention should be given to patients with germline mutations in *BRCA* or *PALB2* given the impact of these genetic changes on response to chemotherapy.

The therapeutic implications of *BRCA* mutations in the palliative management of pancreatic cancer have recently been demonstrated to extend beyond platinum-based chemotherapy. PARP inhibitors have previously shown exciting clinical efficacy in the treatment of *BRCA* mutant-associated breast, ovarian and prostate cancers.^16,17,28^ Golan et al., recently reported the POLO trial, which evaluated olaparib therapy as maintenance treatment for patients with a germline *BRCA1* or *BRCA2* mutation and metastatic pancreatic cancer without progressive disease after at least 16 weeks of platinum-based chemotherapy.^29^ Of 3315 patients screened 247 (7.5%) were found to have a germline mutation in *BRCA1* or *BRCA2* and 154 patients were ultimately randomised. Olaparib maintenance was associated with a significantly longer progression free survival as compared to placebo (7.4 months vs. 3.8 months; *p* *=* 0.004).^29^ Of patients included in the trial who underwent randomisation, nearly 50% were found to have achieved a partial or complete response to platinum-based chemotherapy, consistent with our findings.

Importantly, of patients screened for the POLO trial who were found to have a germline *BRCA* mutation 21.7% had disease progression on platinum-based chemotherapy prior to randomisation and were ineligible for maintenance therapy. Similarly, in our study five mut-positive patients (21%) had progressive disease as best response during treatment with platinum, although we note that three of these patients were treated with platinum in the second-line setting or later. The mechanisms of primary resistance to platinum chemotherapy in *BRCA*-related pancreatic cancer are being actively investigated.

Beyond *BRCA* and *PALB2*, additional germline mutations associated with a DNA damage repair deficient phenotype have been identified in patients with pancreatic cancer and may represent novel therapeutic targets and predictors of response to platinum-based therapy and PARP inhibition.^21,30,31^ The role of these mutations as predictive biomarkers is being actively investigated.

In conclusion, in this small retrospective cohort study we advance our understanding of treatment outcomes in patients with PDAC associated with *BRCA* or *PALB2* germline mutations. Importantly, the high response rate and extended real-world progression free survival in this analysis supports the preferential use of platinum-based therapy for patients with PDAC harbouring a *BRCA* or *PALB2* germline mutation, and supports the need for early germline testing in order to select the best therapy early on in a patient’s treatment course. Further prospective studies are needed to refine the treatment paradigms for this important subset of patients with PDAC.

## Supplementary information

Supplementary information

## Data Availability

No publicly available datasets have been generated as part of this study. All data generated or analysed during this study are included in this published article and its supplementary information files.
