# Supplementary material for: Platinum response characteristics of patients with pancreatic ductal adenocarcinoma and a germline BRCA1, BRCA2 or PALB2 mutation
Source: Br J Cancer. 2019 Dec 2;122(3):333–9. doi: 10.1038/s41416-019-0582-7 (PMC7000723; doi:10.1038/s41416-019-0582-7)
Supplement: Supplementary file 1 — Supplementary information [file 41416_2019_582_MOESM1_ESM.docx]

**Wattenberg *et al* Supplementary information**

Supplementary Figures

**Supplemental figures**

**Supplementary figure 1. Overall survival**

The overall survival was 24.6 months for mut-positive patients and 18.8 months for control patients (*p* = 0.0467).
